# The incidence and risk of developing a second primary esophageal cancer in patients with oral and pharyngeal carcinoma: a population-based study in Taiwan over a 25 year period

**DOI:** 10.1186/1471-2407-9-373

**Published:** 2009-10-20

**Authors:** Kuan-Der Lee, Chang-Hsien Lu, Ping-Tsung Chen, Chunghuang Hubert Chan, Jen-Tsun Lin, Cih-En Huang, Chih-Cheng Chen, Min-Chi Chen

**Affiliations:** 1 Department of Hematology and Oncology, Chang Gung Memorial Hospital at Chiayi, Taiwan, Republic of China; 2 Department of Medicine and Graduate Institute of Clinical Medical Sciences, Chang Gung University, Tao-Yuan; 3 Chang Gung Institute of Technology, Chia-yi Campus, Chiayi, Taiwan, Republic of China; 4 Department of Public Health and Biostatistics Consulting Center, School of Medicine, Chang Gung University, Tao-Yuan, Taiwan, Republic of China

## Abstract

**Background:**

The incidence of oral and pharyngeal (including oral cavity, oropharynx and hypopharynx) carcinoma increases rapidly in Asia and South Pacific because of betel quid chewing. Thus far, large-scale epidemiological studies are not available yet to stratify these patients by their risks of developing a second primary cancer in the digestive tract including esophagus, stomach, colon, and rectum.

**Methods:**

A population-based study was conducted using the database from the Taiwan National Cancer Registry for the period 1979-2003. We quantified standardized incidence ratios (SIRs) and cumulative incidence of second primary cancers among 33,787 patients with initial diagnoses of oral and pharyngeal carcinoma.

**Results:**

Among these four digestive tract organs, the esophagus was the only site of second cancer with excess risk in patients with oral and pharyngeal carcinoma. The incidence and risk of developing a second primary esophageal cancer differed by the site of the primary index tumor, most frequently seen in hypopharyngeal cancer (71/4,218 = 1.68%, SIR = 22.76, 95% CI 17.77-28.70), followed by oropharyngeal cancer (30/3,403 = 0.88%, SIR = 14.29, 95% CI 9.64-20.39) and the least in oral cavity cancer (99/26,166 = 0.38%, SIR = 5.57, 95% CI 4.53-6.78). In addition, the risk was extraordinarily high for patients with a follow-up interval ≤ 1 year and those with first primary cancer diagnosed at age ≤50. These patients may justify more close surveillance.

**Conclusion:**

The present study represents the first population-based study in Asia attempting to stratify the patients of oral and pharyngeal carcinoma by their risk of developing a second esophageal cancer. It helps identify patients at high risk and tailor the application of intense follow-up surveillance to the estimated risk in each individual case.

## Background

The incidence of oral and pharyngeal (including oral cavity, oropharynx and hypopharynx) carcinoma is increasing rapidly in Asia and South Pacific, which includes Taiwan [1,2]. It affected more men than women. In addition to tobacco smoking and alcohol drinking, betel quid chewing has been identified as a significant etiological factor in this area [3]. Oral premalignancies are common in betel quid chewers and about 10% of these undergo malignant transformation. In Taiwan, 17% adult males chewing betel quid, oral and pharyngeal carcinoma has become the malignancy with the fastest increasing incidence [4]. In the past 20 years, its age-standardized incidence rate has increased from 6.04 per 100,000 men in 1986 to 26.36 in 2000, and 32.4 in 2005 [5], strikingly higher than the incidences of the United States and Canada (16 and 13 per 100,000 men in 2004, respectively) [6,7]. Despite modern treatment modalities, the 5-year survival rate of oral and pharyngeal carcinoma has remained essentially unchanged over the past decades, ranging from 40 to 50% [8,9]. This is partly because most patients are often not diagnosed until a late stage and therefore, an oral screening program can not be overemphasized in the high-risk population. The other major cause of death is the high incidence rate of second primary malignancies which impact survival rates the greatest in patients with early-stage disease. The survival after second cancers varies by the site of the second cancer, with esophagus or lung being the worst [10].

The increased risk of second primary esophageal cancer among patients with a first primary oral and pharyngeal carcinoma was reported [11-13], yet there has been limited to single-institutional data, prejudiced by selection bias or small sample size. In 2008, a large-scale study assessed the risk of second primary cancers following a first primary esophageal cancer as well as the risk of esophageal cancer as a second primary [14]. The dataset for analysis was pooled from 13 cancer registries located in Europe, Australia, Canada, and Singapore; of these, only 4.9% cases were in Asia. An excess of second primary esophageal cancer following first primary cancers of the oral and pharyngeal carcinoma was confirmed. To date, there have been controversial opinions regarding routine panendoscopy performed for every patient at the time of initial work-up or in the follow-up [15-19]. Cost-effectiveness is the greatest concern. For cost-saving, the optimal strategy is to identify the subgroup of patients at the highest risk for second primary esophageal cancer. Thus far, large-scale epidemiological studies are not available yet, particularly in the high incidence found of South-East Asia, to stratify oral and pharyngeal carcinoma patients by their risks of developing a second primary cancer in the digestive tract including esophagus, stomach, colon, and rectum. To achieve this goal, we conducted a population-based study using a database from the Taiwan Cancer Registry that included a total of 33,787 subjects with initial diagnoses of oral and pharyngeal carcinoma between 1979 and 2003. This study is, to our knowledge, the largest population-based study from a high-incidence area.

## Methods

### Data sources

We quantified second cancer incidences among 39,118 patients with initial diagnoses of oral and pharyngeal carcinoma, which included the primary cancer originating in the oral cavity (ICD-9:140-145 except 142), oropharynx (including the soft palate, tongue base and tonsil; ICD-9: 146, 149) and hypopharynx (including pypopharynx and pyriform sinus; ICD-9: 148), who were reported to the Taiwan Cancer Registry (TCR) http://crs.cph.ntu.edu.tw between 1 January, 1979 and 31 December, 2003. TCR was founded in 1979 and financially supported by the National Department of Health with the aim of estimating the cancer incidence in Taiwan. It is a population-based cancer registry that covered 22 million people in 2003. Hospitals with > 50 beds were obliged to submit information on newly-diagnosed cancer patients to the TCR, which reimburses the hospitals on the basis of numbers of cases reported in order to reduce the likelihood of under-reporting. All cancer registry databases in the TCR have been systemically converted to International Classification of Diseases, 9th Revision codes [20], and linked with death certificates from the National Death Database. Persons not identified by this process were therefore considered to be alive for the purpose of the current study (passive follow-up). Coding of multiple primaries followed a common set of rules proposed by the International Agency of Cancer Registries (IACR) and the International Agency for Research on Cancer (IARC) [21]. Informed consent was not required because all registry records are anonymous and open to the public.

To assess the age of onset accurately, estimate person-year follow-up and minimize potentially unconfirmed cancer diagnosis in this study cohort, 5,331 patients were excluded from analysis because they met one or more of the following criteria: (1) missing birth dates or unknown gender (522 cases), (2) missing last follow-up date or death status (2,570 cases), (3) second cancer diagnosis or death occurring less than 1 month after the primary oral and pharyngeal cancer (2,195 cases), or (4) age under 20 years old (165 cases). As a result, a total of 33,787 cases (30,176 males and 3,611 females) were included in the analysis. For subsequent risk analyses, the primary cancers were further stratified by anatomic site of origin into oral cavity (ICD-9: 140, 141, 143, 144, 145), oropharynx (ICD-9: 146, 149) and hypopharynx (ICD-9: 148).

### Statistical analysis

To quantify the excess of second malignancies after diagnosis of primary oral and pharyngeal carcinoma, we calculated the standardized incidence ratios (SIRs) [22] and the corresponding 95% confidence intervals (CIs) for all types of second primary cancers. SIRs were taken as the ratio of the observed number (O) of second cancers to the expected number (E), which was obtained by assuming that these persons experienced the same cancer incidence as the corresponding general population. The number of person-years at risk was defined as the number of years from the date of initial primary cancer diagnosis to the date of death, date of last follow-up, date of diagnosis of second primary cancer, or the end of the study period (31 December, 2003), whichever came first. The person-years of observation for each gender, 5-year age group, 5-year period (1979-1983, 1984-1988, 1989-1993, 1994-1998, 1999-2003) and time since entry to the cohort (≤1, 2-5 and >5 years) were multiplied by the incidence rates of cancers for the Taiwanese population. The corresponding products were summed over all ages and calendar years to yield the expected number of second cancer at each site. Confidence intervals of SIRs were based on the assumption of a Poisson distribution of second cancer cases.

Cumulative incidence rates for occurrence of second cancers were calculated in the survivors' cohort, with death treated as a competing risk according to the method of Kalbfleisch and Prentice [23]. Briefly, this method allows for the fact that patients who die are no longer at risk for second cancers, so it differs from the cumulative incidence estimated by the Kaplan-Meier method, which treats competing events as censored at the time they occurred. Gray's test [24] was used to assess the statistical differences of cumulative incidence between two primary index tumors. The survival curves of patients with second esophageal cancer versus other non-esophageal second cancers were calculated by the Kaplan-Meier method and the differences between these two groups were presented by hazard ratio using the Cox proportional hazards model. All statistical tests were two-sided and *P *< 0.05 was considered statistically significant.

## Results

### Patient characteristics

Of the total 33,787 cases (30,176 males and 3,611 females) with oral and pharyngeal carcinoma diagnosed as the first malignancy and complete data available for analysis, which included oral cavity (26,166 cases), oropharynx (3,403 cases) and hypopharynx (4,218 cases), 2,379 cases (7.04%) developed at least one second primary malignancy and 200 cases (0.59%) developed a second primary esophageal cancer during 116,912 person-years of follow-up. The characteristics of the patient population are listed in Table 1. Within this cohort, the average follow-up time was 3.46 years, including 21,704 cases (64%) followed up for at least one year, 4,666 cases (14%) for 5-10 years and 3,144 cases (9%) for >10 years. The mean age at diagnosis of first malignancy was 53.63 (age range 20 to 98) years for the three cancers. For those diagnosed with second primary esophageal cancer, the mean diagnosis age was 56.70 (age range 36 to 83) years with an average interval of 2.52 years (time interval range 0.09 to 12.65) between the diagnosis of the two primary cancers.

**Table 1:** Characteristics of population-based cohort of 33,787 patients diagnosed the first primary cancer as oral and pharyngeal (including oral cavity, oropharyngeal, or hypopharyngeal) carcinoma, 1979-2003.

		**Oral cavity**	**Oropharynx**	**Hypopharynx**
ICD		140, 141, 143, 144, 145	146, 149	148
No. with first primary cancer	All	26,166	3,403	4,218
	M	23,320	2,788	4,068
	F	2,846	615	150
No. who developed a second primary cancer (%)*	All	1,800 (6.88)	232 (6.82)	347 (8.23)
	M	1,654 (7.09)	197 (7.07)	335(8.24)
	F	146 (5.13)	35 (5.69)	12 (8.00)
No. who developed the second esophageal cancer (%)*	All	99 (0.38)	30 (0.88)	71 (1.68)
	M	99 (0.42)	29 (1.04)	69 (1.70)
	F	0 (0)	1 (0.16)	2 (1.33)
Average (± sd) age at diagnosis of first cancer (yrs)		52.70 ± 12.54	54.75 ± 13.17	58.48 ± 11.75
Average (± sd) age at diagnosis of a second cancer (yrs)		56.70 ± 11.87	57.70 ± 12.11	60.12 ± 11.38
Average (± sd) age at diagnosis of the second esophageal cancer (yrs.)		57.59 ± 10.46	51.77 ± 10.52	57.55 ± 10.15
Average (± sd) interval between the first primary and second cancers (yrs.)		3.25 ± 3.51	2. 64 ± 3.47	2.47 ± 3.32
Average (± sd) interval between the first primary and the second esophageal cancer (yrs.)		3.10 ± 2.64	1.24 ± 1.16	2.23 ± 2.76
Average follow-up (yrs)		3.60 ± 4.15	3.39 ± 4.32	2.65 ± 3.80

*percentage out of number with first primary cancer.

sd = standard deviation; yrs = years.

### Risk of second cancer at digestive tracts stratified by the site of primary oral and pharyngeal carcinoma

Second cancer risk in the digestive tract including esophagus, stomach, colon, and rectum, was analyzed in patients with primary oral and pharyngeal carcinoma. Standardized incidence ratios (SIRs) and corresponding 95% confidence intervals (CIs) were calculated by the anatomic site of origin of the primary cancer (Table 2). Among these four digestive tract organs, the esophagus was the only site of second cancer with excess risk in patients with oral and pharyngeal carcinoma. Interestingly, the risk was increased as the primary index tumor was located in proximity to the esophagus, in a descending sequence of hypopharynx (SIR = 22.76, 95% CI 17.77-28.70) > oropharynx (SIR = 14.29, 95% CI 9.64-20.39) > oral cavity (SIR = 5.57, 95% CI 4.53-6.78). The risks of second colon and rectum cancer were similar to the general population, whereas second stomach cancer was decreased in oral cavity cancer patients (SIR = 0.58, 95% CI 0.39-0.84), suggesting that it occurred less frequently than expected.

**Table 2:** Risk for esophagus (ICD-9: 150), stomach (ICD-9: 151), colon (ICD-9:153) and rectum (ICD-9: 154) as the second primary cancer site among 33,787 oral/pharyngeal cancer (ICD-9: 140-149, except 142 and 147) patients.

**First primary cancer site (ICD-9)**			**Esophagus**			**Stomach**			**Colon**			**Rectum**		
						
	**Sex**	**OPY**	**O**	**E**	**SIR** **(95% CI)**	**O**	**E**	**SIR** **(95% CI)**	**O**	**E**	**SIR** **(95% CI)**	**O**	**E**	**SIR** **(95% CI)**
			
Oral cavity (140-1, 143-5)	All	90644	99	17.73	**5.57** **(4.53-6.78)**	28	48.02	**0.58** **(0.39-0.84)**	39	41.63	0.93 (0.66-1.28)	35	36.15	0.97 (0.67-1.34)
	M	77527	99	17.25	**5.74** **(4.66-6.99)**	28	42.82	**0.65** **(0.43-0.95)**	31	34.68	0.89 (0.61-1.27)	29	30.90	0.94 (0.63-1.35)
	F	13117	0	0.48	0 (NA)	0	5.20	0 (NA)	8	6.95	1.15 (0.50-2.27)	6	5.25	1.14 (0.42-2.49)
Oropharynx (146,149)	All	11303	30	2.11	**14.29** **(9.64-20.39)**	2	6.51	0.31 (0.03-1.11)	0	5.63	0 (NA)	3	4.77	0.63 (0.13-1.83)
	M	8474	29	2.01	**14.43** **(9.66-20.72)**	0	5.48	0 (NA)	0	4.25	0 (NA)	3	3.73	0.80 (0.16-2.35)
	F	2829	1	0.09	11.11 (0.15-61.82)	2	1.03	1.94 (0.22-7.01)	0	1.38	0 (NA)	0	1.04	0 (NA)
Hypopharynx (148)	All	11212	71	3.12	**22.76** **(17.77-28.70)**	6	9.40	0.64 (0.23-1.39)	7	7.18	0.97 (0.39-2.01)	8	6.18	1.29 (0.56-2.55)
														
	M	10721	69	3.09	**22.33** **(17.37-28.26)**	6	9.17	0.65 (0.24-1.42)	6	6.87	0.87 (0.32-1.90)	8	5.95	1.34 (0.58-2.65)
														
	F	491	2	0.02	**100** **(11.23-361.05)**	0	0.23	0 (NA)	1	0.30	3.33 (0.04-18.55)	0	0.23	0 (NA)

Bold indicates statistical significance.

OPY = observed person years; SIR = standardized incidence ratio; O = observed numbers of second primary cancers; E = expected numbers of second primary cancers; CI = confidence interval; NA = not assessable.

### Risk of second esophageal cancer stratified by follow-up interval after oral and pharyngeal carcinoma

To explore the latency of development of the second esophageal cancer, the standardized incidence ratio estimates were stratified by interval since the first diagnosis of oral and pharyngeal cancers (Table 3). The entire follow-up period was categorized into 3 intervals: ≤1 year, 1-5 years and >5 years. The risk of developing a second esophageal cancer peaked during the first-year of follow up, with the descending sequence of hypopharynx (SIR = 81.13, 95% CI 59.57-110.88) > oropharynx (SIR = 75.00, 95% CI 47.12-116.41) > oral cavity (SIR = 18.18, 95% CI 12.62-25.47), and decreased with follow-up time but remained elevated for 5 years after diagnosis of the first primary cancer.

**Table 3:** Risk for second esophageal cancer by follow-up interval after the diagnosis of oral/pharyngeal cancer.

First primary cancer site (ICD-9)	Follow-up time (yrs)	OPY	O	E	SIR (O/E)	95% CI
Oral cavity (140-1,143-145)	≤1	10352	34	1.87	**18.18**	**(12.62-25.47)**
	1-5	25205	45	4.91	**9.16**	**(6.69-12.27)**
	> 5	55087	20	10.96	**1.82**	**(1.11--2.82)**
						
Oropharynx (146, 149)	≤1	1449	21	0.28	**75.00**	**(47.12-116.41)**
	1-5	2862	9	0.53	**17.31**	**(7.83-32.56)**
	> 5	6992	0	1.30	0	(NA)
						
Hypopharynx (148)	≤1	2119	43	0.53	**81.13**	**(59.57-110.88)**
	1-5	3005	20	0.77	**25.97**	**(15.76-39.87)**
	> 5	6088	8	1.82	**4.40**	**(1.89 -- 8.66)**

Bold indicates statistical significance.

OPY = observed person years; SIR = standardized incidence ratio; O = observed numbers of second esophageal cancer; E = expected numbers of second esophageal cancer; CI = confidence interval;

NA = not assessable; yrs = years.

### Age trend of second esophageal cancer

To study the trend of the second esophageal cancer with age at initial diagnosis of the oral and pharyngeal carcinoma, we stratified their standardized incidence ratios (SIRs) according to three age groups (<50, 50-60 and >60) (Table 4). The occurrence of second esophageal cancer exhibited a strong trend with the onset age of oral and pharyngeal carcinoma. The risk was much higher in younger patients, particularly those diagnosed before 50 years of age, the SIR was drastically high, in a descending order of hypopharynx (SIR = 71.53, 95% CI 43.67-110.48) > oropharynx (SIR = 46.49, 95% CI 25.40-78.01) > oral cavity (SIR = 11.01, 95% CI 7.83-15.05). For those aged >50, their SIRs also remained significantly high.

**Table 4:** Risk for second esophageal cancer by age at initial onset among 33,782 patients with oral/pharyngeal cancer.

First primary cancer site (ICD-9)	Age (yrs)	OPY	O	E	SIR (O/E)	95% CI
Oral cavity (140-1, 143-145)	≤50	39031	39	3.54	**11.01**	**(7.83-15.05)**
	50-60	24591	30	5.65	**5.31**	**(3.58-7.58)**
	> 60	27022	30	8.54	**3.51**	**(2.37-5.02)**
						
Oropharynx (146, 149)	≤50	4333	14	0.30	**46.49**	**(25.40-78.01)**
	50-60	2869	11	0.62	**17.73**	**(8.84-31.73)**
	> 60	4101	5	1.18	**4.22**	**(1.36-9.85)**
						
Hypopharynx (148)	≤50	2479	20	0.28	**71.53**	**(43.67-110.48)**
	50-60	3300	26	0.83	**31.22**	**(20.39-45.75)**
	> 60	5433	25	2.01	**12.47**	**(8.07-18.41)**

Bold indicates statistical significance.

OPY = observed person years; SIR = standardized incidence ratio; O = observed numbers of second esophageal cancer; E = expected numbers of second esophageal cancer; CI = confidence interval; yrs = years.

### Risk of second esophageal cancer stratified by calendar year at diagnosis of oral and pharyngeal carcinoma

SIRs stratified for calendar year at diagnosis of the first primary cancer of the oral cavity, oropharynx and hypopharynx were calculated for second primary esophageal cancer across the diagnostic periods 1979-1993 and 1994-2003 (Table 5). The SIR demonstrated a trend of increasing excess risk of second esophageal cancer in oropharyngeal carcinoma after 1994 but remained relatively stable in oral cavity and hypopharyngeal carcinomas.

**Table 5:** Risk for second esophageal cancer by calendar period at diagnosis of oral/pharyngeal cancer.

First primary cancer site (ICD-9)	Calendar year	OPY	O	E	SIR (O/E)	(95% CI)
Oral cavity (140-1, 143-145)	1979-1993	20031	27	8.94	**8.94**	**(5.89-13.01)**
	1994-2003	70613	72	4.89	**4.89**	**(3.83-6.16)**
						
Oropharynx (146, 149)	1979-1993	2930	2	4.55	4.55	(0.51-16.41)
	1994-2003	8373	28	16.87	**16.87**	**(11.21-24.38)**
						
Hypopharynx (148)	1979-1993	3052	18	25.35	**25.35**	**(15.02-40.07)**
	1994-2003	8160	53	21.99	**21.99**	**(16.47-28.77)**

Bold indicates statistical significance.

OPY = observed person years; SIR = standardized incidence ratio; O = observed numbers of second esophageal cancer; E = expected numbers of second esophageal cancer; CI = confidence interval.

### Cumulative incidence rates of all second cancers versus the second esophageal cancer

**Figure 1:**
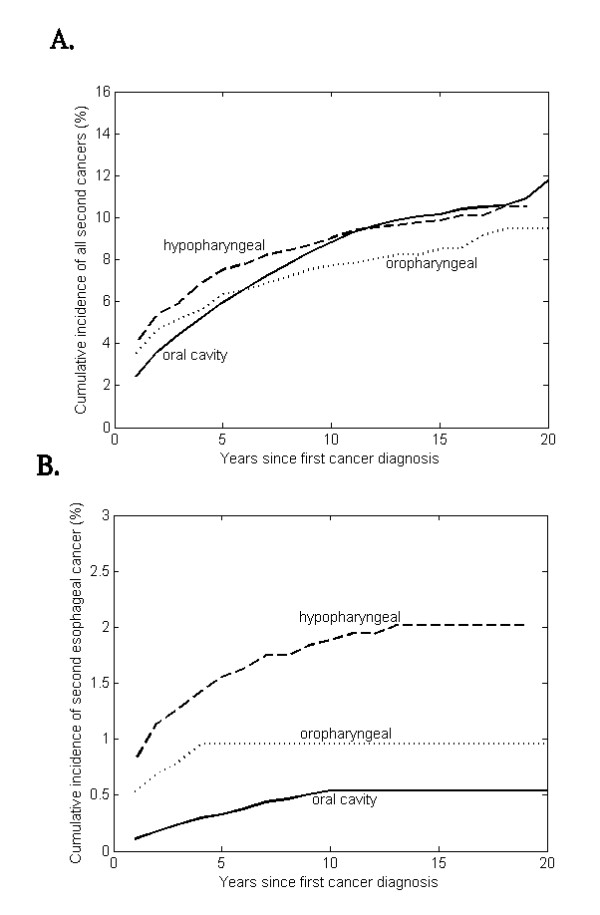
**Cumulative incidence rates of (A) all the second cancers, and (B) second primary esophageal cancer for a total of 33,787 patients with primary oral/pharyngeal carcinoma**.

The estimated overall risk of developing all types of second cancers after primary oral and pharyngeal carcinoma in the survivors' cohort was calculated with death treated as a competing risk (Figure 1A), while the estimated overall risk of developing a second esophageal cancer after primary oral and pharyngeal carcinoma in the survivors' cohort was calculated with death and non-esophageal cancers treated as competing risks (Figure 1B).

The overall cumulative risks of all second cancers at 5, 10, 15 years after the first primary cancer diagnosis were estimated to be 6.13%, 9.02%, and 10.36%, respectively, for oral cavity cancer; 6.48%, 7.87%, and 8.66%, respectively, for oropharyngeal cancer; and 7.57%, 9.07%, and 9.94%, respectively, for hypopharyngeal cancer. There was no risk plateau and the cumulative incidences over time did not differ each other among oral cavity, oropharynx and hypopharynx (all *P*- values > 0.05), indicating that they were at equivalent risk for developing second cancers without specified.

When the second cancer was restricted to esophageal cancer, the overall cumulative risks at 5, 10, 15 years after primary cancer were estimated to be 0.34%, 0.55%, and 0.55%, respectively, for oral cavity cancer; 0.98%, 0.98%, and 0.98%, respectively, for oropharyngeal cancer; and 1.57%, 1.90%, and 2.02%, respectively, for hypopharyngeal cancer. The risk plateau was seen in all the cumulative incidence curves. The cumulative incidence curves for developing a second esophageal cancer were statistically different when compared each other (all *P*-values < 0.01), in a sequence of hypopharynx > oropharynx > oral cavity. The trend in Figure 1B was in consistent with the results observed in Table 2 and Table 3.

### Overall survival of the oral and pharyngeal carcinoma patients

**Figure 2:**
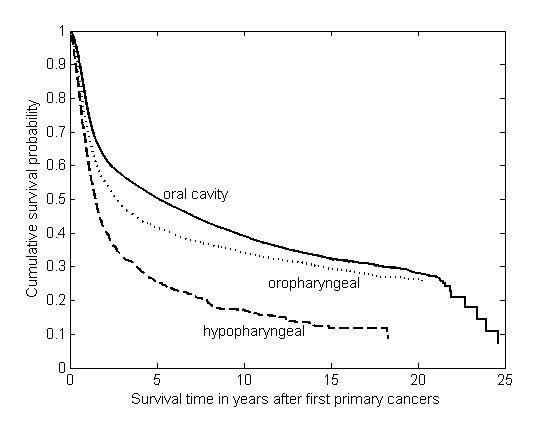
**Kaplan-Meier survival curves of all the patients stratified by index tumor site in the oral cavity (26,166 cases), oropharynx (3,403 cases) and hypopharynx (4,218 cases)**.

The overall 5-year survival rate for all oral and pharyngeal carcinoma patients in our cohort was 50.2 ± 0.002% with a median survival of 3.93 ± 0.08 years. For stratification by tumor site, the 5-year survival rate was 50%, 41%, and 28% for oral cavity, oropharynx, and hypopharynx, with the median survival time of 5.11, 2.66 and 1.46 years, respectively (Figure 2). The survivals were significantly different each other (all *P*-values < 0.001), in which hypopharyngeal cancer was associated with the worst survival, followed by oropharyngeal cancer, when compared to oral cavity cancer.

### Survival time after second esophageal cancer versus non-esophagus second cancers

**Figure 3:**
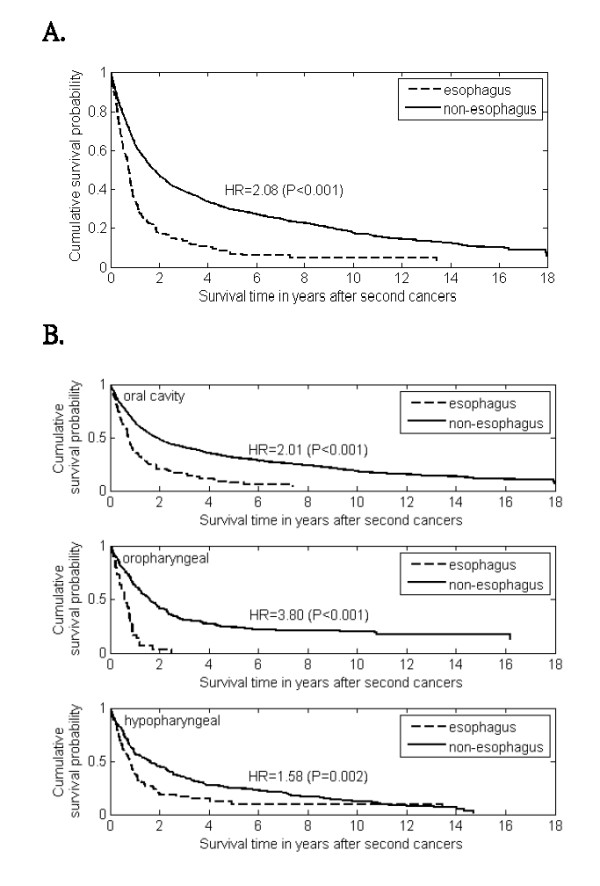
**The survival curves of second esophageal cancer versus non-esophageal second cancers for (A) all the patients with oral/pharyngeal carcinoma; and (B) patients stratified by index tumor site**.

The survival time after diagnosis of the second esophageal cancer as compared to other non-esophagus second cancers was calculated by the Kaplan-Meier method. The results suggest that irrespective of primary site, second esophageal cancer had a shorter survival than other non-esophagus second cancers (Hazard ratio, HR= 2.081, *P*-value < 0.001) (Figure 3A). For specific primary tumor site, the median survival after developing a second esophageal cancer was 0.73 ± 0.06, 0.60 ± 0.09, and 0.81 ± 0.09 years for oral cavity, oropharynx, and hypopharynx, respectively, whereas the median survivals after developing other non-esophagus second cancers were 1.85 ± 0.10, 1.63 ± 0.20, and 1.60 ± 0.24 years for oral cavity, oropharynx, and hypopharynx, respectively. The results revealed that second esophageal cancer had a shorter survival than other non-esophageal second cancers (HR = 2.01, 3.80, 1.58 for oral cavity, oropharynx and hypopharynx, respectively, all *P*-values ≤ 0.002) (Figure 3B).

## Discussion

Second primary malignancies, especially esophageal cancer, are known to be one of the major causes of treatment failure in patients with oral and pharyngeal carcinoma, particularly those with early stage. An early diagnosis of asymptomatic esophageal cancer can prolong survival and enables treatment by endoscopic mucosal resection [25]. However, in our study, the incidence of developing a second primary esophageal cancer was only 0.59%. Because of the relatively small proportion, routine panendoscopy as the initial evaluation and follow-up examination for every oral and pharyngeal carcinoma patient has remained controversial in Taiwan. For cost-effectiveness, it would be more logical to identify patients at the highest risk and tailor the application of intensive follow-up screening to the estimated risk in each individual case. We found the incidence and risk of developing a second primary esophageal cancer differed according to the site of the primary index tumor, most frequently seen in hypopharyngeal cancer (71/4,218= 1.68%, SIR= 22.76, 95% CI 17.77-28.70), followed by oropharyngeal cancer (30/3,403= 0.88%, SIR= 14.29, 95% CI 9.64-20.39) and the least in the oral cavity cancer (99/26,166= 0.38%, SIR= 5.57, 95% CI 4.53-6.78). The longer the patients survive the first cancer, the greater their risk of developing a second primary. Comparing to oropharyngeal cancer and oral cavity cancer with 41% and 50% five-year survival rate respectively, hypopharyngeal cancer has a relatively low five-year survival of only 28% and thus less opportunity to develop a second primary cancer. However, it was noted that hypopharyngeal cancer had the highest risk of developing a second esophageal cancer.

In this analysis, the risk was extraordinary high for patients with initial cancer onset at age ≤50 and those with follow-up interval ≤ 1 year. A multicentric study published by Chuang et al [14] also showed the similar findings. These patients may justify more close surveillance and periodic panendoscopies. The increased risk for young patients could be explained by a possible inherited genetic susceptibility to cancer in addition to environmental risk factors. There is evidence that genetically predisposed individuals tend to develop a second primary malignancy following head and neck cancer [26]. However, there are still some limitations in this study. We could not exclude the possibility that the observed excess risk in the first year of follow-up might be due to more frequent examinations (surveillance bias), or a misclassification of the local spread out of the primary tumor to esophagus as a second primary.

The etiologic factors for developing a subsequent esophageal cancer in oral and pharyngeal carcinoma patients remain to be defined. Alcohol consumption and cigarette smoking are major risk factors in oral and pharyngeal carcinoma as well as esophageal cancer [26,27]. In Taiwan, betel quid chewing is also associated with higher risk of oral cavity and esophageal cancer [28], and an interaction between cigarette, alcohol and betel quid use on esophageal cancer risk has been reported [29].

Field cancerization [30] can explain in part the development of multiple tumors by shared common risk factors, where the carcinogenic effects of alcohol, tobacco and betel nut may simultaneously act on the entire mucosa of mouth, pharynx and aerodigestive tract to trigger the development of multiple cancers that are independent of each other. As to other agent, the association of human papilloma virus (HPV) with oropharyngeal cancer (45%), particularly Waldeyer's tonsillar ring (60%), has been reported [31], yet its etiologic role in the development of esophageal cancer is not conclusive, particularly in different geographic areas [32,33]. Interestingly, studies from China [34,35] have reported relatively high percentages of HPV-positive esophageal cancer cases when compared to reports from Western countries. In contrast, HPV was not found to be associated with esophageal cancer in Taiwan [36] and Korea [37]. In addition to environmental factors such as tobacco, alcohol, betel and HPV, there was an age trend for esophageal cancer as a second primary, with SIR higher in younger onset patients than older onset patients, suggesting that genetic predisposition may play a role.

Regardless of primary site, the median survival after developing a second esophageal cancer did not exceed one year. Furthermore, among those with a second cancer, those with an esophageal cancer had a risk of death over 2-fold compared to those with a non-esophageal second cancer. The dismal prognosis related to a second esophageal cancer may be attributable to late diagnosis and in some patients, the inability to receive aggressive therapy because of therapy for the first malignancy. The reduction of risk for gastric cancer in the oral cavity cancer patients is an unanswered question arising from our study. In a single-institute study with 1,138 cases of malignancies of the head and neck in Japan [38], the risk of second gastric cancer was even higher than that of second esophageal cancer. This is in contrast to our findings that no excess risk was observed with non-esophageal cancers (i.e. stomach, colon and rectum). The discrepancy was intriguing and could be due to selection bias in a single hospital or geographic variations. Further studies are needed to elucidate their associations.

## Conclusion

The results of our study observed strong associations of first primary oral and pharyngeal carcinoma with second primary esophageal cancer, which had a great impact on survival. This study represents the first population-based study in Asia attempting to stratify the patients of oral and pharyngeal carcinoma by their risk of developing a second esophageal cancer. Such knowledge will aid in the appropriate selection of high-risk patients for a follow-up surveillance program, and can be very useful for some countries of Asia and the South Pacific where the incidence rates of oral and pharyngeal carcinoma are high, but implementation of such program is not easy due to limited resources.

## Competing interests

The authors declare that they have no competing interests.

## Authors' contributions

KDL and CHL conceived the study design and performed data collection. MCC contributed in statistical analysis. KDL and MCC wrote the manuscript with inputs from CHL, PTC, CHC, JTL, CEH and CCC. All authors read and approved the final manuscript.

## Pre-publication history

The pre-publication history for this paper can be accessed here:

http://www.biomedcentral.com/1471-2407/9/373/prepub
